# The Effects of a Fasting Mimicking Diet on Skin Hydration, Skin Texture, and Skin Assessment: A Randomized Controlled Trial

**DOI:** 10.3390/jcm12051710

**Published:** 2023-02-21

**Authors:** Jessica Maloh, Min Wei, William C. Hsu, Sara Caputo, Najiba Afzal, Raja K. Sivamani

**Affiliations:** 1 Integrative Skin Science and Research, Sacramento, CA 95815, USA; 2 L-Nutra, Inc., Plano, TX 75024, USA; 3 School of Medicine, University of California-Davis, Sacramento, CA 95817, USA; 4 Department of Dermatology, University of California-Davis, Sacramento, CA 95816, USA; 5 College of Medicine, California Northstate University, Elk Grove, CA 95757, USA; 6 Pacific Skin Institute, Sacramento, CA 95815, USA

**Keywords:** fasting-mimicking diet, skin hydration, skin texture

## Abstract

Diet and nutrition have been shown to impact dermatological conditions. This has increased attention toward integrative and lifestyle medicine in the management of skin health. Emerging research around fasting diets, specifically the fasting-mimicking diet (FMD), has provided clinical evidence for chronic inflammatory, cardiometabolic, and autoimmune diseases. In this randomized controlled trial, we evaluated the effects of a five-day FMD protocol, administrated once a month for three months, on facial skin parameters, including skin hydration and skin roughness, in a group of 45 healthy women between the ages of 35 to 60 years old over the course of 71 days. The results of the study revealed that the three consecutive monthly cycles of FMD resulted in a significant percentage increase in skin hydration at day 11 (*p* = 0.00013) and at day 71 (*p* = 0.02) relative to baseline. The results also demonstrated maintenance of skin texture in the FMD group compared to an increase in skin roughness in the control group (*p* = 0.032). In addition to skin biophysical properties, self-reported data also demonstrated significant improvement in components of mental states such as happiness (*p* = 0.003) and confidence (0.039). Overall, these findings provide evidence for the potential use of FMD in improving skin health and related components of psychological well-being.

## 1. Introduction

The utilization of integrative medicine and lifestyle interventions in the management of various health concerns has gained significant interest in recent years. A lifetime prevalence of 35–69% for the use of complementary medicine was found in patients who seek dermatological treatments [1]. Among the various modalities of integrative medicine, diet and nutrition play a particularly important role. By addressing the underlying dietary and nutritional factors that may contribute to skin concerns, dietary interventions in dermatology may offer a unique inside-out approach to care [2].

The relationship between diet, weight, and dermatological disease has been demonstrated in the literature. For example, high-glycemic load diets are associated with hyperinsulinemia which, in turn, can contribute to acne through increases in inflammation and androgen-mediated increases in sebum production [3]. A randomized controlled trial in those with acne found that intervention with a low-glycemic load diet improved insulin sensitivity and reduced total inflammatory lesion counts compared to control [4]. Furthermore, psoriasis has been associated with increased adiposity, with excess adipose tissue that contributes to a pro-inflammatory state [5]. A study was conducted to investigate the role of weight reduction on psoriasis with the use of a low-calorie, protein-based diet [5]. After four weeks, a significant improvement was found in measures of quality of life, pain, itch, and the extent and severity of psoriasis [5].

Fasting has gained popularity in recent years. Fasting is conventionally defined as the abstinence of food and/or beverages for periods of time, usually for several hours of a day or lasting for days [6]. A recent prospective observational study evaluated the effects of fasting during the Islamic observance of Ramadan in individuals with psoriasis [7]. Ramadan is a month-long observance associated with engaging in intermittent daily fasting, where all food and beverages are avoided from dawn to sunset. This type of fasting was found to be associated with a significant reduction in the extent and severity of psoriasis symptoms [7]. Additionally, there were notable improvements in metabolic markers, including a significant decrease in fasting plasma glucose and triglycerides and a significant increase in HDL cholesterol [7]. These findings indicate that intermittent fasting during Ramadan may have potential therapeutic benefits for individuals with psoriasis.

An alternative method of fasting that has gained attention in recent years is the Fasting Mimicking Diet (FMD). This approach involves the consumption of a specifically formulated, calorie-restricted nutrition regimen with a customized macronutrient composition, ratio, and quantity over a 5-day period. It aims to evade nutrient sensing by cells and to elicit a similar physiological response to fasting. The FMD has been shown to be safe and efficacious in both animal and human studies for various parameters and conditions [8]. A study that assessed the effects of FMD on mice revealed that after lifelong feeding with cycles of FMD, mice had lower insulin and glucose signaling, lower visceral fat and reduced pro-inflammatory cytokines [9]. There is also evidence to suggest that FMD can significantly decrease circulating levels of growth factor, contributing to its potential anti-cancer and anti-aging properties [9].

The benefits of the FMD have also been demonstrated in human clinical trials, including reductions in body weight and visceral adiposity, insulin growth factor-1 (IGF-1), serum glucose and biomarkers associated with aging [10,11]. Moreover, research has found that FMD can support gut health by promoting markers of regeneration and improving intestinal healing under inflammation-driven stress [12]. The FMD has also been shown to consistently reduce intestinal inflammation, increase stem cell number, and promote the growth of protective gut microbiota, such as *Lactobacillaceae* and *Bifidobacteriacea* while modifying the presence of other microbes in mice [12].

A number of studies have examined the effects of fasting-mimicking diets in various conditions, including inflammatory bowel disease, diabetes, cardiovascular disease, cancer, multiple sclerosis, Alzheimer’s disease, and depression in animal models and human studies [11,12,13,14,15,16,17,18,19,20,21,22,23]. However, there are currently no human clinical trials assessing the effect of an FMD in dermatology. This is the first randomized controlled trial to assess the efficacy of a five-day FMD protocol done once a month for three cycles on facial skin parameters such as skin hydration and skin roughness in a group of women over the course of 71 days.

## 2. Methods 

### 2.1. Subject Enrollment

A total of 45 female participants, ranging in age from 35 to 60 years old, were recruited for the study. This protocol was approved by the Advarra Institutional Review Board (24 November 2021). All participants provided written consent prior to participation. Inclusion criteria for participants were as follows: good general health and social well-being, Fitzpatrick skin type I-VI, and mild to moderate scores for parameters on the global face (fine dry lines, roughness, uneven skin tone, and lack of radiance). Exclusion criteria included a known allergy to any component of the meal kit, breastfeeding, pregnancy or planned pregnancy during the study, history of gastric bypass, or history of skin cancer. Having a health condition and/or pre-existing or dormant dermatologic disease on the face (ex: psoriasis, rosacea acne, eczema, seborrheic dermatitis, and severe excoriations) that the investigator deemed inappropriate for participation or deemed to interfere with the outcome of the study also served as an exclusion.

### 2.2. Randomization and Blinding

The participants were randomized into two groups, with 24 individuals in the intervention group and 21 subjects in the control group. In order to ensure the integrity of the study, the study products were dispensed by a separate individual, not involved in the investigation or evaluation process. Additionally, the research participants and the research staff responsible for dispensing the study products were instructed to refrain from discussing study products with the investigator or other evaluator(s). This allowed the evaluators to be blinded during evaluations.

### 2.3. Participant Instructions and Group Interventions

All participants were instructed to maintain their regular exercise and physical activity habits, avoid prolonged sun exposure and use of tanning beds or sunless tanning products. Participants were also asked to continue their regular use of cosmetics, makeup, and sunscreen and to refrain from the use of new facial products for the duration of the study.

The intervention group was provided with ProLon^TM^, a fasting-mimicking diet product, to be consumed for five consecutive days, with the first usage beginning on day 1, followed by an additional usage on day 30, and on day 60. ProLon was intended to replace usual meals during the five-day period. Following the five-day period, participants were instructed to keep meals light on day 6 and resume normal eating habits on day 7. The composition of the test product and daily meal plans are detailed in Table 1. The control group did not receive any specific diet and was instructed to maintain their habitual diet for the duration of the study. Participants were evaluated during five visits throughout the study, with the first evaluation on day 0, the second evaluation on day 11, the third evaluation on day 30, the fourth evaluation on day 60, and the fifth and final evaluation on day 71.

Diaries were provided to each participant in the intervention group at the beginning of the study to record their consumption of the test product. Participants were also asked to document any additional food or beverage items consumed during the five-day period beyond the provided meal kit and to note any changes in eating patterns between the fasting cycles.

### 2.4. Skin Assessments

Prior to any skin assessments, it was ensured that all subjects did not have any makeup or topical products on their face and were allowed to acclimate to ambient conditions within the clinic for at least 15 min. The designated rooms were maintained at a temperature range of 68–75 °F and a relative humidity range of 35–65%.

Individuals in both groups completed a 22-item 5-point Likert scale self-assessment questionnaire to evaluate various skin parameters, including texture, hydration and skin tone. In order to measure skin hydration, Corneometer (CK Electronic, Köln, Germany) measurements were taken on the center of each subject’s right cheek. Antera 3D (Miravex, Dublin, Ireland) imaging was performed on the skin surface with multi-directional illumination and computer-aided reconstruction of the skin surface. Texture (Ra, or mean roughness) was analyzed using the Antera 3D CS software.

### 2.5. Statistics

Sample size estimation was based on prior similar dietary intervention studies [11,24]. Demographic and clinical characteristics were described according to the study group using means and standard deviations for continuous variables and count with frequencies for categorical variables. A descriptive statistical summary was provided for all efficacy grading parameters, bio instrumentation (Corneometer) measurements, Antera image analysis, and self-assessment questionnaires with baseline response data. The descriptive statistical summary included the sample mean, median, SD, MIN, and MAX of scores/values at all applicable time points. Questionnaires were tabulated, and the frequency and percentage of all response options were reported for each question and time point. For questionnaires without baseline response data, a binomial (sign) test was performed to test if the proportion of the combined designated favorable responses is equal to the combined designated unfavorable responses for each applicable question. Corneometer and Antera 3D imaging measurement results were tested using paired t-test and 2 sample *t*-test for the change from the baseline and between groups, respectively. Self-assessment measures were tested by the Wilcoxon signed rank test and Wilcoxon rank sum test to assess the change from baseline and between groups, respectively. All statistical tests were 2-sided at significance level alpha = 0.05 unless specified otherwise. Statistical analyses were performed using SAS software version 9.4 (SAS Statistical Institute).

## 3. Results

**Figure 1:**
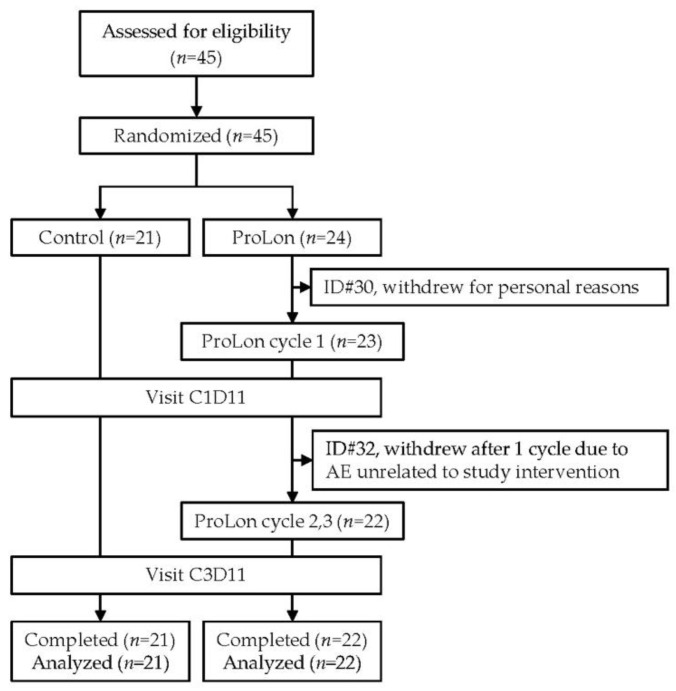
CONSORT (Consolidated Standards of Reporting Trials) flow diagram. C1D11, cycle 1, day 11; C3D11, cycle 3, day 11.

Out of the 45 participants that were screened for eligibility, all 45 met the criteria and were subsequently enrolled in the study. The 21 subjects randomized into the control group all completed the study, whereas 22 out of the 24 subjects randomized into the treatment group completed the study. A CONSORT diagram is presented in Figure 1 to illustrate the flow of participants throughout the study.

### 3.1. Demographics

The demographics of the study population are outlined in Table 2.

### 3.2. Skin Hydration

**Figure 2:**
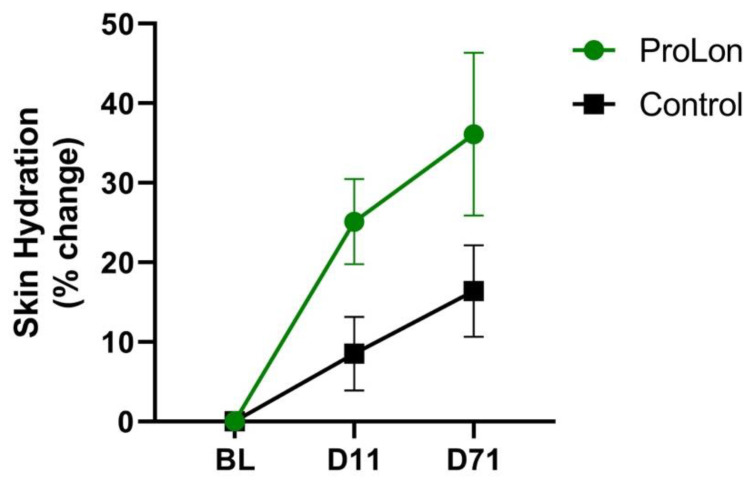
Percent change in skin hydration using corneometer measurements at baseline (BL), day 11 (D11), and day 71 (D71).

When assessing the percent increase in skin hydration, there was a significantly greater increase in the treatment group (25.1%) relative to the control (8.52%) at day 11, which corresponds to five days after completing the first ProLon cycle and after the resumption of subjects’ habitual diet (*p* = 0.024). Additionally, there was a statistically significant increase in percent change for skin hydration at both day 11 and day 71 in the treatment group relative to baseline (*p* = 0.00013 and *p* = 0.02, respectively). However, in the control group, a statistically significant increase was only observed at day 71 compared to the baseline (*p* = 0.0098) (Figure 2).

### 3.3. Skin Roughness

**Figure 3:**
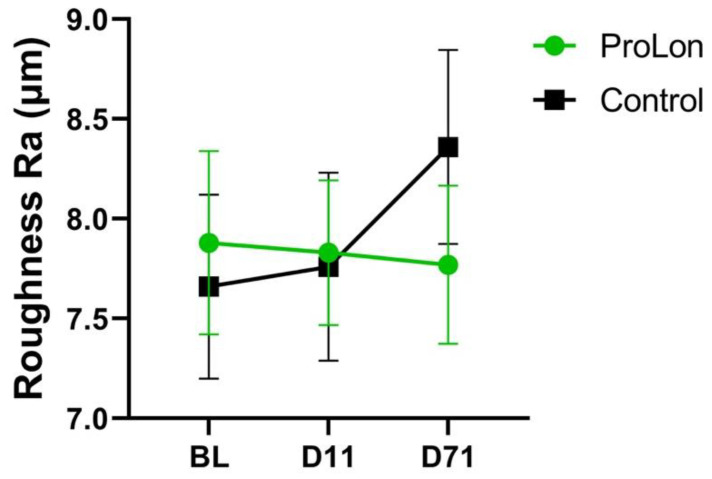
Skin Roughness Changes. The skin roughness was followed over the course of the study period in both the treatment and control groups.

Skin texture, as determined by mean roughness (Ra), did not exhibit a significant change in the treatment group at day 11 or at day 71 relative to baseline. However, mean roughness was found to increase in the control group significantly at day 71 relative to baseline (*p* = 0.032) (Figure 3).

### 3.4. Subjective Outcomes

**Figure 4:**
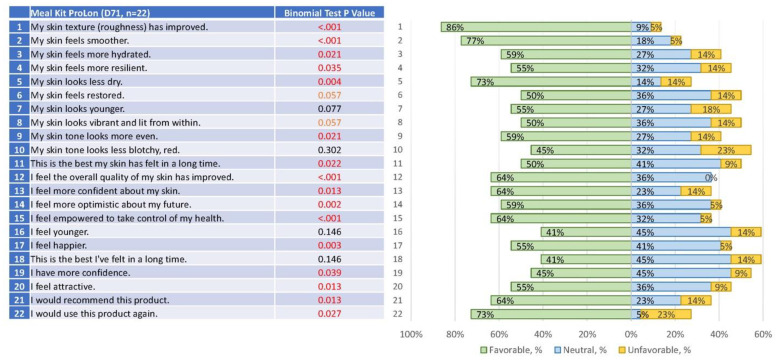
Self-reported Skin Assessments. The *p*-values for the comparisons on self-reported outcomes are shown (**left**) in relation to the responses (**right**).

After completion of 3 ProLon cycles, subjects in the intervention group reported a significant improvement in various aspects of skin health, including (1) skin texture (*p* < 0.001), (2) smoothness (*p* < 0.001), (3) hydration (*p* = 0.021), and (9) skin tone evenness (*p* = 0.021), along with other skin improvements presented in Figure 4. In addition, there were significant improvements in some aspects of mood and self-perception (Figure 4). Specifically, subjects in the treatment group reported significant improvements in feelings of (17) happiness (*p* = 0.003), (19) confidence (0.039), (15) empowerment to take control of their health (*p* < 0.001), and (14) optimism about the future (*p* = 0.002).

Overall, the ProLon intervention was well tolerated. The adverse effects reported during the study were not deemed by the investigators to be serious events and were not deemed to be associated with the intervention. They included one case of each of the following: left-hand paresthesia, left leg pain, arthralgia, pyelonephritis, and knee ligament rupture, all of which resolved.

## 4. Discussion

This study aimed to evaluate the effect of fasting-mimicking diet with monthly ProLon on the skin. The results indicate that FMD has a beneficial effect on objective measures of skin parameters such as hydration and texture. Additionally, there was an improvement in multiple self-reported outcomes related to skin appearance and general well-being.

Although FMD has not been previously studied for its effects on skin health, there is support for the use of caloric restriction in improving skin anatomy and function. Caloric restriction with fasting has demonstrated improvement in multiple skin properties, including skin barrier function in both mice and humans [25]. Studies have also suggested that caloric restriction may improve the appearance of wrinkles and decrease the presence of oxidative stress [26,27].

The potential mechanisms by which FMD may exert its effects on the skin are multifaceted. Fasting has been shown to initiate comprehensive cellular and systemic reprogramming in organisms in response to starvation conditions. Biogerontological research in the past 30 years has linked prolonged nutrient deprivation with the downregulation of pro-growth signaling and activation of cellular protection mechanisms, which may have implications for the amelioration of disease-associated factors and the delay of aging [8,28]. The FMD was specifically designed to mimic the effects of water-only fasting and had been shown to induce anti-oxidative stress in cells [29,30], dampen the mTOR-S6K signaling pathway, activate autophagy [31,32,33], promote stem cell-based regenerations in multiple tissues [12,14,34], augment the gut microbiome [12], and reduce risk factors associated with age-related diseases [11]. Further research on the use of period fasting interventions such as FMD may reveal it to be a cost-effective and feasible component of an integrative approach to skin health.

Another potential mechanism by which FMD may impact skin health is through the gut-skin axis. Research suggests that subsequent cycles of FMD may reduce intestinal inflammation and stimulate protective members of the gut microbiome, such as *Lactobacillaceae* and *Bifidobacteriacea* [12]. These specific members of the gut microbiota have been found to be relevant to skin health. For example, children with eczema have been found to have less gut colonization by *Bifidobacterium* and *Lactobacillus* strains relative to healthy control [35,36]. Furthermore, in an animal study, oral supplementation with *Bifidobacterium breve* B-3, a member of the *Bifidobacteriacea* family, has been found to protect against UV-induced changes in transepidermal water loss and changes in skin hydration [37]. However, additional research will be needed to better understand the relationship between fasting-mediated shifts in the gut microbiome and changes in skin outcomes.

In this study, the self-reported data was found to be consistent with the objective skin biophysical measurements. For example, the participants in the treatment group reported improvement in skin hydration, which was in alignment with the objective measure of the studies. This suggests that the extent of skin improvement was to a noticeable degree, which may imply clinical relevance. Furthermore, the results from self-assessments suggest that the FMD impacted aspects of mental well-being and self-esteem, demonstrated by improvements in the feeling of happiness, confidence, and attractiveness. Previous studies investigating FMD have also found improvements in mental states. In one study, patients with depression that received both FMD and psychotherapy had an improvement in self-esteem and psychological quality of life compared to a group receiving psychotherapy alone [23]. It is interesting to note that our FMD study reported improvement in aspects of self-esteem in generally healthy subjects.

This trial serves as a pilot study to demonstrate the effects of FMD on skin health, and the parameters assessed warrant further investigation with a larger sample size. Furthermore, because this study was done with a healthy population, future research should focus on individuals with a skin condition to better understand the use of the FMD in the setting of dermatological disease. Additionally, future research can expand the study population to include male participants. With evidence suggesting improvements in aspects of mental well-being, further research on the FMD and skin health should incorporate validated questionnaires for mood to better understand the skin-mind axis in the context of fasting.

## 5. Conclusions

The results of this study are the first to investigate the role of fasting-mimicking interventions and, specifically, FMD for skin health and appearance in a randomized controlled trial. Skin hydration and skin roughness were found to be supported with the use of the FMD, along with self-reported improvements in components of mental wellness, including happiness, confidence, and optimism. Overall, FMD is found to be a promising intervention in the realm of integrative and lifestyle medicine for skin health. Future studies should evaluate this intervention in individuals with skin diseases to better understand the mechanisms and effects of FMD in clinical dermatology settings.

## Figures and Tables

**Table 1:** Test products to be used on day 1, day 30, and day 60.

	Day 1	Day 2	Day 3	Day 4	Day 5
**Breakfast**	– Nut-based bar – Herbal tea – Algal oil—(2 packets)	– Nut-based bar – Herbal tea	– Nut-based bar – Herbal tea	– Nut-based bar – Herbal tea	– Nut-based bar – Herbal tea – Algal oil—(1 packet)
**Lunch**	– Vegetable soup – NR-1 * (2 packs) – Kale crackers – Olives	– Vegetable soup – NR-1 * (1 pack) – Olives	– Vegetable soup – NR-1 * (1 pack) – Kale crackers	– Vegetable soup – NR-1 * (1 pack) – Olives	– Vegetable soup – NR-1 * (1 pack) – Kale crackers
**Afternoon**	– Herbal tea – Nut-based bar	– Herbal tea – Olives	– Herbal tea	– Herbal tea – Olives	– Herbal tea
**Dinner**	– Vegetable soup – Chocolate crisp bar	– Vegetable soup – Chocolate crisp bar	– Vegetable soup	– Vegetable soup – Chocolate crisp bar	– Vegetable soup
**Daily**		L-Drink	L-Drink	L-Drink	L-Drink

* NR-1 Capsules (Vegetable Powder with Vitamins and Minerals).

**Table 2:** Study population demographics.

	ProLon (Treatment)	Control
N	22	21
Age, yr (SD)	49.5 (8.0)	48.7 (7.7)
BMI (SD)	28.8 (4.9)	26.0 (3.8)
Asian	7	3
White	10	12
Hispanic	3	4
Black	2	2
Fitzpatrick type		
II	4	4
III	11	14
IV	6	2
V	1	1
Skin type		
Normal	5	6
Combination	7	10
Dry	9	5
Oily	1	0
Sensitive skin	12	10
Reactive skin	0	1

## Data Availability

Data is available upon request to the corresponding author.
